# Deep Cascade of Convolutional Neural Networks for Quantification of Enlarged Perivascular Spaces in the Basal Ganglia in Magnetic Resonance Imaging

**DOI:** 10.3390/diagnostics14141504

**Published:** 2024-07-12

**Authors:** Seunghye Chae, Ehwa Yang, Won-Jin Moon, Jae-Hun Kim

**Affiliations:** 1 Medical Research Institute, Samsung Medical Center, Seoul 06351, Republic of Korea; lloves2743@gmail.com; 2 School of Medicine, Sungkyunkwan University, Seoul 06351, Republic of Korea; 3 Department of Radiology, Konkuk University Medical Center, School of Medicine, Konkuk University, Seoul 05030, Republic of Korea; 4 Department of Radiology, Samsung Medical Center, Sungkyunkwan University School of Medicine, Seoul 06351, Republic of Korea

**Keywords:** enlarged perivascular spaces, deep learning, image enhancement, quantification

## Abstract

In this paper, we present a cascaded deep convolution neural network (CNN) for assessing enlarged perivascular space (ePVS) within the basal ganglia region using T2-weighted MRI. Enlarged perivascular spaces (ePVSs) are potential biomarkers for various neurodegenerative disorders, including dementia and Parkinson’s disease. Accurate assessment of ePVS is crucial for early diagnosis and monitoring disease progression. Our approach first utilizes an ePVS enhancement CNN to improve ePVS visibility and then employs a quantification CNN to predict the number of ePVSs. The ePVS enhancement CNN selectively enhances the ePVS areas without the need for additional heuristic parameters, achieving a higher contrast-to-noise ratio (CNR) of 113.77 compared to Tophat, Clahe, and Laplacian-based enhancement algorithms. The subsequent ePVS quantification CNN was trained and validated using fourfold cross-validation on a dataset of 76 participants. The quantification CNN attained 88% accuracy at the image level and 94% accuracy at the subject level. These results demonstrate significant improvements over traditional algorithm-based methods, highlighting the robustness and reliability of our deep learning approach. The proposed cascaded deep CNN model not only enhances the visibility of ePVS but also provides accurate quantification, making it a promising tool for evaluating neurodegenerative disorders. This method offers a novel and significant advancement in the non-invasive assessment of ePVS, potentially aiding in early diagnosis and targeted treatment strategies.

## 1. Introduction

Perivascular spaces (PVSs), also known as Virchow–Robin spaces, are fluid-filled spaces surrounding penetrating blood vessels in the brain [1], essential for transporting cerebrospinal fluid (CSF) and clearing waste products [2,3]. While normally microscopic and invisible, PVS can expand and become visible in MRI due to aging or neurological disorders, indicating obstruction of fluid flow or disruption in the blood–brain barrier. Current understanding is that PVS enlargement refers to the obstruction of fluid flow due to protein and cellular debris, causing fluid to accumulate [2]. This phenomenon can be understood as trapping unremoved cerebrospinal fluid in the subpial or interpial space [4]. The expansion of PVS associated with fluid accumulation signifies more than just structural changes but also substantial functional disruption in the fluid transport mechanisms within the brain [5], which leads to various neurological disorders [6].

Several studies have identified ePVS as a potential biomarker for small vessel pathology and glymphatic dysfunction in brain disorders such as dementia [7], stroke [8], multiple sclerosis [9], Parkinson’s disease [10], and insomnia [11]. The frequency of ePVS is used as an imaging indicator for cerebral small vessel disease (CSVD) and is associated with cognitive impairment and dementia. Enlarged PVS in the basal ganglia is related to decreased information processing speed [12], highlighting the link between CSVD and cognitive decline. Additionally, the number of ePVSs in the basal ganglia correlates with subcortical vascular cognitive impairment and vascular dementia [13,14]. Studies suggest that ePVS is considered a biomarker for small vessel disease (SVD), and given the progression of SVD to vascular dementia, the association between ePVS and vascular dementia is reasonable [6]. Moreover, the ePVS burden in Alzheimer’s disease patients is correlated with glymphatic dysfunction indicators [15]. Therefore, the need for ePVS markers is emphasized for the clinical diagnosis of dementia. 

MRI is essential for the visual assessment of ePVS due to its ability to provide high-resolution images and contrast with surrounding tissue [16]. ePVS appears as low-intensity on T1-weighted MRI and fluid-attenuated inversion recovery (FLAIR), but shows bright signal intensity on T2-weighted MRI, where it shows clearly defined boundaries. Despite this, manual annotation of ePVS is challenging due to its variable size and shape, as well as the limitations of 1.5 T and 3 T MRI scanners. Additionally, the presence of numerous ePVS in a single scan complicates accurate visual assessment. Raters often need to zoom in/out or scroll through slices to distinguish ePVSs from similar-appearing brain lesions, such as lacunar infarcts or small white matter lesions [17,18]. Current clinical studies rely on visual scoring systems, where expert raters count the number of ePVSs within a region of interest (ROI) [19] or use grading systems like Potter’s five-point scale [20]. These manual methods are time-consuming and subject to variability, highlighting the need for automated ePVS quantification to improve accuracy and efficiency in clinical settings.

Recent studies have focused on automated methods to address the limitations of manual ePVS assessment. Rashid et al. used a fully convolutional neural network with 7 T T2-weighted MRI for efficient ePVS segmentation [21], but 7 T MRI is not commonly used in clinical settings where 1.5 T or 3 T MRI is standard. Therefore, researchers have explored automated quantification methods using image enhancement technology on lower-resolution MRIs. For example, Uchiyama et al. used white top-hat filtering on 1.5 T MRI for lesion enhancement [22], Yang et al. applied Haar transformation on 3 T MRI [23], Park et al. proposed an automated ePVS segmentation method based on randomized Haar features [24], and Ballerini et al. used the Frangi filter for individual ePVS segmentation [25]. However, these methods involve complex heuristic parameter tuning. To overcome these issues, Jung et al. introduced a deep learning-based approach that eliminates the need for additional processing, using densely connected deep CNNs to generate enhanced images accurately [26]. Their study was still limited to 7 T MRI and focused on enhancement rather than quantification.

This study aims to improve the accuracy and efficiency of ePVS assessment by proposing a cascaded deep convolutional neural network (CNN) framework to enhance and quantify ePVS in T2-weighted MRI, specifically using the clinically prevalent 3 T MRI.

Research Question:

1. Can deep learning-based enhancement improve ePVS visibility on T2-weighted MRI?
2. How effective are deep learning-based enhancement approaches in quantifying ePVSs compared to traditional methods?

This study intends to answer these questions through comparative experiments, demonstrating superior quantification performance compared to algorithm-based enhancement techniques.

## 2. Materials and Methods

### 2.1. Dataset

The dataset used in this study consists of brain MRI scans of a total of 76 patients from a prospective BIG-VARISTA cohort (KCT0003181) provided by Konkuk University Medical Center (FEB 2018.02 to May 2020). Participants provided written informed consent. This study was approved by the institutional review board (IRB-21-06-036). It was carried out in accordance with the principles of the Declaration of Helsinki. Table 1 summarized the demographic information of the patients.

All the images were acquired using a 3.0 T Siemens Skyra scanner with a 20-channel coil. The protocol includes 2D T2WI, 3D T1-weighted MRI, 3D FLAIR MRI, and 3D SWI. The T2-weighted scan was acquired using an axial turbo spin-echo sequence with a TR/TE of 4450 ms/81 ms, matrix size of 384 × 384, in-plane resolution of 0.573 × 0.573 mm, slice thickness of 5 mm, and 28 slices. The T1-weighted scan was obtained using a magnetization-prepared rapid gradient echo (MPRAGE) sequence with a repetition time (TR)/echo time (TE) of 2300 ms/2.98 ms, inversion time of 900, matrix size of 256 × 256, in-plane resolution of 1 × 1 mm, slice thickness of 1 mm, and 192 slices. The FLAIR 3D sequence used a TR/TE of 5000 ms/393 ms, inversion time of 1800 ms, matrix size of 256 × 256, in-plane resolution of 1 × 1 mm, slice thickness of 1 mm, and 196 slices.

### 2.2. Labeling for ePVS

The images in our dataset were visually graded by a single expert rater with 22 years of neuroimaging experience (MWJ). The neuroradiologist rater, blinded to clinical information, visually assessed ePVSs in T1-weighted, T2-weighted, and FLAIR MR images. The neuroradiologist analyzed three consecutive slices covering basal ganglia regions, separately assessing the left and right sides. Ratings were based on Potter’s grading system: 0 for no ePVSs, 1 for 1–10 (mild), 2 for 11–20 (moderate), 3 for 21–40 (frequent), and 4 for over 40 (severe) ePVSs. The final rating of ePVS for the subject was determined as the highest score among the six scores from three contiguous slices and each hemisphere. Interobserver agreement of PVS was reported in our previous study. In that study, two neuroradiologists (with 22 years and 8 years of experience) studied 20 healthy controls’ T2 images from a separate dataset, and the inter-agreement using the intraclass correlation coefficient (ICC) was 90.1% (95% confidence interval: 0.8–0.952) [19]. 

### 2.3. Deep Cascade of Convolutional Neural Networks

**Figure 1:**
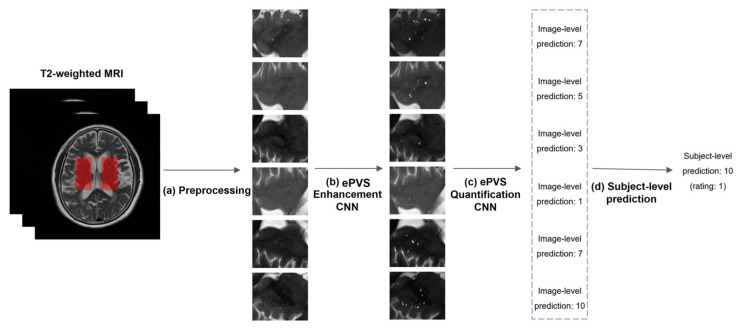
The flowchart for a deep cascade of CNNs for the assessment of ePVS at image level and subject level. (**a**) Preprocessing. Six 2D images were extracted from T2-weighted MRI in the left and the right basal ganglia regions across three slices. Each 2D image was resized and normalized. (**b**) ePVS enhancement CNN. The 2D image was enhanced to have a clear visibility of ePVS using CNN. (**c**) ePVS quantification CNN. The number of ePVSs from the enhanced image was predicted using a CNN (image-level prediction). (**d**) Subject-level prediction. The maximum operation is applied to the 6 predicted values for each subject.

Figure 1 shows the overall flowchart for a deep cascade of CNNs for the assessment of ePVSs at image level and subject level.

#### 2.3.1. Preprocessing

**Figure 2:**
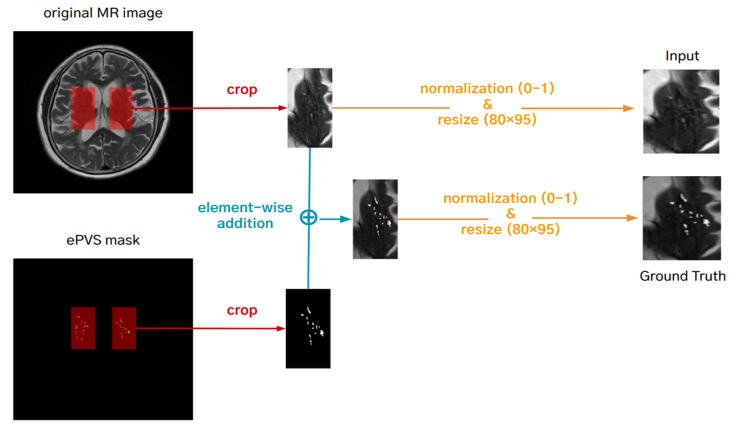
Preprocessing for making a ground truth for the deep learning-based enhancement network.

The 2D images were created from the T2-weighted MRI in the left and right basal ganglia regions across three slices, resulting in six 2D images per subject. Each 2D image was resized to 80 × 96 using bilinear interpolation, which produces smoother images by reducing artifacts. The resizing to 80 × 96 pixels was determined to balance computational efficiency with the preservation of critical anatomical details. This size is small enough to reduce computational load and training time, yet large enough to retain essential structural details of the ePVS. This balance ensures that downsampling by the neural network does not degrade image quality, allowing for the accurate detection and quantification of ePVSs. We used 16-bit medical images. To improve the convergence rate of the optimization algorithm, these values were normalized to the [0, 1] range. The ground truth for the ePVS enhancement network was generated by element-wise adding of the normalized T2-weighted MRI and binary ePVS mask, enhancing the ePVS contrast, and then normalizing the enhanced image to [0, 1] (Figure 2). The ground truth (the number of ePVSs) for the quantification of ePVS network was also normalized to [0, 1] by dividing the maximum number of ePVSs (in our case: 48). 

#### 2.3.2. Deep Convolutional Neural Network for ePVS Enhancement

**Figure 3:**
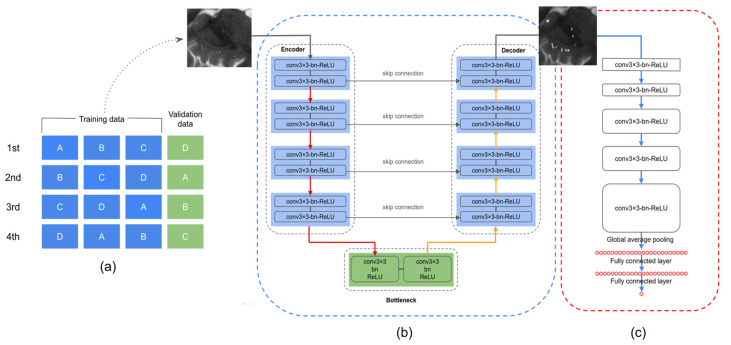
(**a**) Schematic representation of the 4-fold cross-validation process. (**b**) Deep CNN for ePVS enhancement. (**c**) Deep CNN for ePVS quantification.

The goal of this stage is to obtain images with clear visibility of ePVS using deep learning, which can provide more useful input data for the second stage of the framework, the quantification of ePVSs. We used the U-Net architecture [27], designed to transform the input image into an enhanced output image (Figure 3b). The U-Net consists of a downsampling pathway and an upsampling pathway, connected by skip connections to preserve spatial information.

Model Architecture:

- Downsampling Pathway: This pathway captures coarse features. It consists of two 2D convolutional layers at each level, followed by batch normalization and ReLU activation functions, and a max-pooling layer to reduce spatial dimensions. We retain the output of each downsampling level before max-pooling for use in the upsampling pathway.
- Upsampling Pathway: This pathway reconstructs the image with enhanced features. It uses transpose convolution (deconvolution) layers to upsample the image. Each upsampled image tensor is concatenated with the corresponding output from the downsampling pathway via skip connections. These connections help capture both fine-grained details and coarse features effectively.

Modification for Enhancement:

We used the U-Net architecture without any structural changes. However, to transform the task from segmentation to image regression, we changed the loss function to mean squared error (MSE). This modification allows for the model to focus on enhancing the visibility of ePVSs within the entire image rather than segmenting it.

Overall, this U-Net model regresses input images to output images with improved clarity of ePVSs. The resulting images with enhanced ePVSs serve as valuable input data for the subsequent stage of our framework: the ePVS quantification network.

#### 2.3.3. Deep Convolutional Neural Network for ePVS Quantification

The CNN architecture for the quantification of ePVSs is shown in Figure 3c. The purpose of this stage is to count the number of ePVSs from the enhanced images obtained in the previous stage.

Model Architecture:

- Convolutional Layers: The model consists of 5 convolutional layers. The first two layers use 64 filters, the next two use 128 filters, and the final layer uses 256 filters. Each convolutional layer is followed by batch normalization and ReLU activation functions to stabilize and improve learning.
- Global Average Pooling Layer: This layer reduces each feature map to a single value, preserving the most important information while reducing dimensionality.
- Fully Connected Layer: The features extracted by the convolutional layers are fed into a fully connected layer, which outputs a scalar value representing the predicted number of ePVSs.

This CNN model is designed to extract essential features related to the number of ePVSs from the input image and predict the number of ePVSs accurately. 

Workflow and Clarifications: 

To clarify the overall workflow of our methodology, we provide the following summary: 

MRI images are preprocessed and then fed into a U-Net model for ePVS enhancement. The U-Net model uses mean squared error (MSE) as the loss function to focus on improving the visibility of ePVSs in the images. The enhanced images from the U-Net model are then input into a CNN designed for ePVS quantification. This network, consisting of multiple convolutional layers, a global average pooling layer, and a fully connected layer, accurately predicts the number of ePVSs.

### 2.4. Implementation

The models were implemented using TensorFlow 2.6 on three 1080Ti GPUs with an Intel(R) Core(TM) i7-6850K CPU*12 @ 128 GB(16 GB*8) RAM and a 64-bit operating system. All networks were trained using the adaptive moment estimation (Adam) optimizer with a learning rate of 1 × 10^−4^. The mean squared error (MSE) was used as the loss function for the ePVS enhancement network, and the mean absolute error (MAE) for the ePVS quantification network. The following augmentation techniques were used during training: *x*- and *y*-axis flips, scaling (0.9 to 1.1), and rotation (−15 degrees to 15 degrees). The batch size was set to 45. The ePVS enhancement network was trained for 500 epochs, and the ePVS quantification network was trained for 300 epochs.

### 2.5. Evaluation

To evaluate the proposed methods, a 4-fold cross-validation approach was applied to a collection of 76 data samples (Figure 3a). During each ‘fold’ of the validation process, a single part (representing 25% of the data) was set aside for testing the model’s performance, while the remaining three parts (cumulatively representing 75% of the data) were used to train the model. This process was repeated four times, with each of the four parts being used as the testing set once, as shown in Figure 3a. For evaluation of the ePVS enhancement network, the CNR was determined. CNR measures the clarity of the ePVS regions compared to their surrounding areas. It is calculated using the following formula:

$$CNR=\frac{\left|\mu_{signal1}-\mu_{signal2}\right|}{\sigma_{noise}}$$

where:

- $\mu_{signal1}$ is the mean signal intensity in the ePVS region.
- $\mu_{signal2}\;$is the mean signal intensity in the surrounding area.
- $\sigma_{noise}$ is the standard deviation of the noise, measured in the background of the slice.

To evaluate the ePVS quantification network, we first converted the normalized output from the network quantification into the number of ePVSs by multiplying it by the maximum number of ePVSs. To compare the estimated ePVS count from the deep learning model with the actual observed counts, the MAE was calculated between ground truth and model outputs. MAE was specifically selected for its direct, clinically interpretable assessment of prediction errors, measured in the same units as ePVSs, and for its resilience to the effects of outliers, which can skew MSE results. Moreover, to determine model accuracy, ePVS ratings were assigned using Potter’s grading system, with categories ranging from 0 to 4. We trained the deep learning model on a per-slice basis, which led us to report the model’s accuracy at the image level. However, given that in clinical settings an individual subject was evaluated using multiple MRI slices, we adopted the highest rating score among six parts corresponding to each subject to report the accuracy of subject level. Additionally, we measured the intraclass correlation coefficient (ICC) among entire subjects to evaluate the reliability of our method.

$$MSE=\frac{1}{n}\sum_{i=1}^{n}={\left(y_{i}-{\hat{y}}_{i}\right)}^{2}$$

$$MAE=\frac{1}{n}\sum_{i=1}^{n}=|y_{i}-{\hat{y}}_{i}|$$

where

- $y_{i}$ is the actual observed count of ePVS.
- ${\hat{y}}_{i}$ is the predicted count of ePVS from the model.
- n is the number of samples.

## 3. Results

### 3.1. Data (MRI, Basal Ganglia ROI, and ePVS ROI)

**Figure 4:**
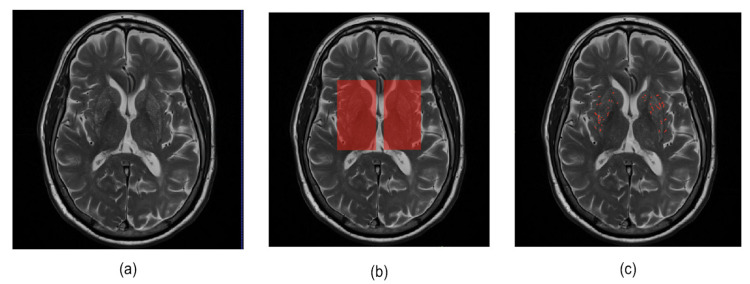
Illustration of example data used in this study. (**a**) Original T2-weighted MRI, (**b**) basal ganglia ROI mask, and (**c**) ePVS segmentation mask.

In this study, we conducted experiments using a dataset of 456 images from 76 participants. The basal ganglia ROI mask is used to acquire six 2D images from T2-weighted MRI and ePVS segmentation masks corresponding to the basal ganglia regions. These images were then employed in a deep learning model for the enhancement of ePVSs. Table 2 shows the characteristics of the ePVS rating score distribution for a total of 456 images extracted from the basal ganglia area. In addition, the images obtained by adding the ePVS segmentation mask (0, 1) to each 2D image (0–1 range) are used as the output for the deep learning model for enhancement of ePVS. Figure 4 provides examples of T2-weighted MRI, basal ganglia ROI, and ePVS ROI images that we used in our study.

### 3.2. Results for Enhancement of ePVS

**Figure 5:**
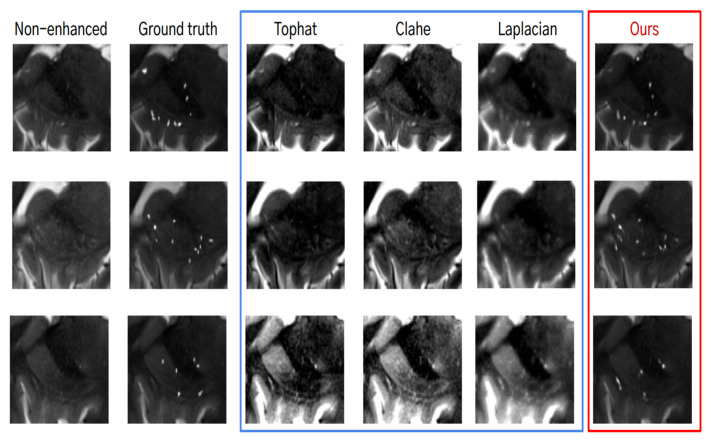
Qualitative analysis for the algorithm-based enhancement (blue box) and the deep learning-based enhancement of ePVS (red box).

For the evaluation of the deep learning-based enhancement of ePVS, the outputs were qualitatively and quantitatively compared with algorithm-based enhancement methods. Figure 5 shows examples of non-enhanced images, images with algorithm-based image enhancement techniques applied (Tophat [28], Clahe [29], Laplacian [30]), and images with deep learning-based enhancement methods. When using algorithm-based image enhancement techniques, we observed that the signal increased not only for ePVS but also for the other organs. On the other hand, the enhanced images using the deep learning method exhibited an increased signal for ePVS, not for the other organs. This indicates that the deep learning method selectively enhances the ePVS signal, improving visibility without introducing noise from other structures.

From the quantitative analysis, we found that the deep learning-based enhancement method showed the highest contrast-to-noise ratio (CNR) of 113.77 ± 15.14 compared with the algorithm-based enhancement methods, which showed CNRs of less than 50 (Table 3). This significant improvement in CNR suggests that the deep learning-based method is highly effective in enhancing ePVS, providing clearer and more distinguishable images.

### 3.3. Results for Quantification of ePVS

**Figure 6:**
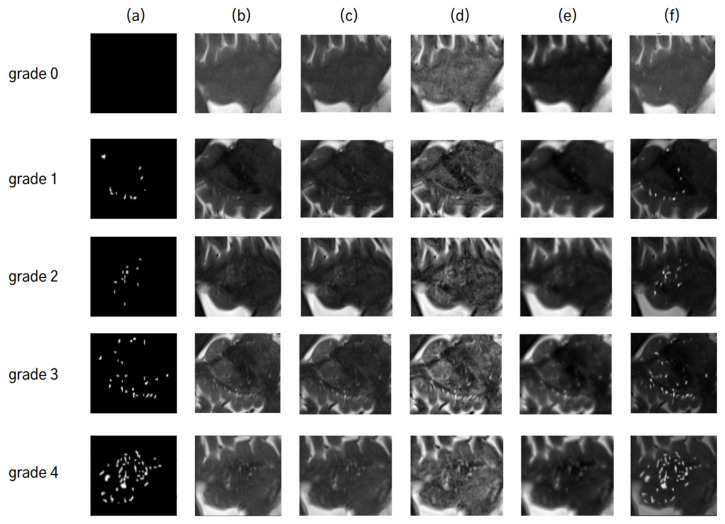
Comparative analysis of ePVS quantification using different image enhancement techniques: (**a**) represents the ePVS segmentation mask, (**b**) is the non-enhanced image, and images (**c**–**f**) are enhanced using Tophat, Clahe, Laplacian, and deep learning techniques, respectively. For each grade from 0 to 4, the predicted grades by each technique are as follows: For grade 0, a predicted grade of ‘1’ is obtained from non-enhanced image (**b**) and images enhanced by techniques in Tophat (**c**), Clahe (**d**), and deep learning (**f**), whereas a predicted grade of ‘2’ comes from Laplacian (**e**). For grade 1, a predicted grade of ‘1’ comes from (**c**,**f**), while a predicted grade of ‘2’ is given by (**b**,**d**,**e**). For grade 2, a predicted grade of ‘2’ is common among (**b**,**d**–**f**) with an exception for (**c**) predicting a lower grade of ‘1’. For grade 3, (**b**,**e**,**f**) predict an accurate grade of ‘3’, while others predict it as lower at ‘2’. For grade 4, only the enhanced image using deep learning (**f**) predicts this highest level accurately with ‘4’.

**Figure 7:**
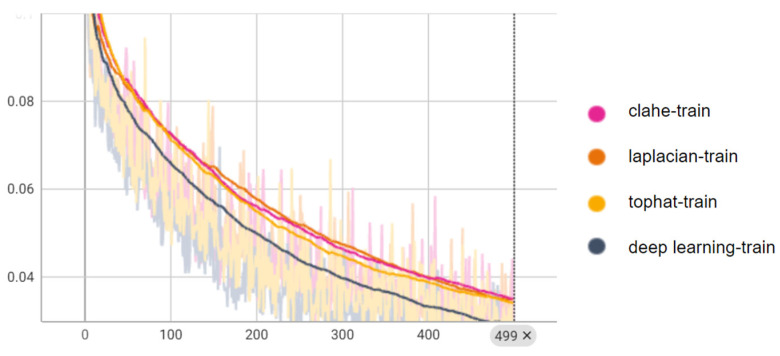
Training curves quantification networks, showing the MAE loss metrics over epochs for both algorithm-based and deep learning-based methods. These curves demonstrate the convergence behavior and learning efficiency of the models, highlighting the advantages of deep learning in achieving higher performance.

To examine how the various enhancement methods influence the quantification of ePVS, we trained the five deep learning models for the quantification of ePVSs according to the various enhanced images (Figure 6). When quantifying ePVSs using the deep learning-based enhanced images, we achieved the highest performance, with 88% accuracy at the image level and 94% accuracy at the subject level, outperforming all other methods (Table 4). As a baseline, quantifying ePVSs using non-enhanced images resulted in 74% accuracy at the image level and 72% accuracy at the subject level. Comparatively, using the Clahe method for deep learning-based quantification resulted in similar accuracy to the baseline, with a 1% lower accuracy for the Laplacian method and a 1% higher accuracy for the Tophat method at the image level. At the subject level, the Tophat method showed similar accuracy, the Clahe method exhibited a 3% higher accuracy, and the Laplacian method had a 9% lower accuracy compared to the baseline model. Interestingly, despite the Laplacian method having the second-highest performance in enhancing ePVS, it showed the worst performance in quantifying ePVSs at both image and subject levels. Conversely, the Clahe method demonstrated the second highest performance in quantifying ePVSs at the subject level, even though it had the worst performance in enhancing ePVS. This discrepancy highlights the importance of using a robust enhancement method tailored for accurate quantification. Additionally, the intraclass correlation coefficient (ICC) using the deep learning-based enhanced images shows high reliability of 90.6% (95% confidence interval: 0.89–0.92) at the image level and 95.2% (95% confidence interval: 0.92–0.97) at the subject level. These high ICC values indicate that the deep learning-based enhancement method not only improves the visibility of ePVSs but also enhances the reliability and consistency of ePVS quantification. These results suggest that deep learning-based enhancement significantly improves both the visual and quantitative assessment of ePVSs compared to traditional algorithm-based methods. To further illustrate the training process and convergence of both algorithm-based and deep learning-based enhancement methods, we have included the training curves in Figure 7. These curves highlight the efficiency and robustness of the deep learning approach compared to traditional methods. These results suggest that deep learning-based enhancement significantly improves both the visual and quantitative assessment of ePVS compared to traditional algorithm-based methods.

## 4. Discussion

In the present study, we proposed a cascaded deep CNN for the assessment of ePVS in the basal ganglia region in T2-weighted MRI comprising deep CNN-based enhancement and quantification stages. To evaluate the enhancement of ePVS, we compared the CNR with various algorithm-based enhancement methods. The deep CNN-based quantification network was evaluated using mean absolute error (MAE). Our deep learning models were evaluated using a fourfold cross-validation approach. 

Our deep CNN-based ePVS enhancement network presents two advantages over conventional algorithm-based enhancement methods. First, our method allows for us to generate enhanced ePVS images without the need for additional heuristic parameters, streamlining the process by eliminating manual fine-tuning. Secondly, our method selectively enhances only ePVS regions, avoiding the global enhancement of entire images seen with traditional methods. This selective enhancement ensures accuracy and clarity in visualizing ePVSs without unnecessary alterations to other image components. 

To assess the impact of our image enhancement method based on deep learning, we applied the quantification network to non-enhanced images, algorithmically enhanced images, as well as deep learning-based enhanced images. Interestingly, despite an increase in CNR scores when using images enhanced by algorithm-based techniques compared to non-enhanced images, the quantification results were similar or even worse. One possible explanation for this could be that the enhancement of not only ePVS but also unnecessary areas during image enhancement may have impacted the quantification. In contrast, our deep learning-based enhancement method led to higher accuracies: reaching up to 88% at the image level and an impressive 94% at the subject level. This means that our deep learning-based approach improved accuracy by a margin of 14% for images and by a margin of 22% for subjects. These outcomes demonstrate that our deep learning-based approach for the enhancement of ePVSs significantly aids in the quantification of ePVSs.

These results are consistent with previous research that highlights the challenges of using algorithm-based methods for image enhancement in medical imaging. However, our study extends this research by demonstrating how deep learning-based image enhancement techniques can significantly aid in quantification. Specifically, our focus on ePVSs in the basal ganglia, a region closely associated with vascular dementia, aims to improve both image clarity and quantification accuracy. Improved ePVS assessment can aid in diagnosing and monitoring neurological disorders where ePVS is a relevant biomarker, such as dementia, stroke, and Parkinson’s disease. This enhanced diagnostic capability could lead to earlier detection and more targeted treatment strategies.

Several limitations should be acknowledged in our study. Firstly, our deep learning models were trained and tested on a relatively small dataset from a single institution, consisting of data from only 76 participants. This small sample size may limit the statistical power and the robustness of the model, potentially leading to overfitting and reduced generalizability. Validation across diverse acquisition parameters from different institutions is required to ensure the robustness and applicability of our findings in broader clinical settings. This limitation highlights the need for multi-center studies to ensure the generalizability of our findings. Secondly, our models were developed solely using T2-weighted MRI, and future research could explore the inclusion of various MRI modalities such as T1-weighted MRI and FLAIR to potentially enhance the accuracy of ePVS quantification. Lastly, our ePVS quantification model was focused on the basal ganglia region, and further investigations could expand our methodology to cover the entire brain. Future research should address the limitations and explore the broader applicability of this methodology to different MRI modalities and brain regions. Additionally, incorporating advanced techniques such as attention mechanisms into the U-Net architecture could further improve the model’s performance. Attention mechanisms have been shown to enhance the ability of neural networks to focus on relevant features, which could lead to more accurate and efficient enhancement and quantification of ePVSs. Evaluating the impact of these mechanisms on our model would be a valuable extension of this study. 

## 5. Conclusions

Our cascaded deep CNN model demonstrated superior performance, achieving 88% accuracy at the image level and an impressive 94% accuracy at the subject level compared to CNN models using Tophat-, Clahe-, and Laplacian-based enhanced algorithms. These results highlight the effectiveness of our deep learning-based approach for ePVS enhancement and quantification in the basal ganglia region on T2-weighted MRI.

Our key findings and their significance include enhanced image quality, where the deep CNN-based enhancement method significantly improved the contrast-to-noise ratio (CNR) of ePVS images, surpassing traditional algorithm-based methods. This selective enhancement leads to clearer and more accurate visualization of ePVSs. Additionally, our quantification network achieved higher accuracy levels, demonstrating that enhanced images from the deep learning model can be effectively used for reliable ePVS quantification. The use of a fourfold cross-validation approach in our study indicates the robustness and consistency of our model’s performance. 

The potential clinical impact of our cascaded deep CNN model is significant for diagnosing neurodegenerative disorders related to the basal ganglia, including small vessel pathology, glymphatic dysfunction, and Alzheimer’s disease. Improved ePVS assessment can aid in early diagnosis, monitoring disease progression, and developing targeted treatment strategies, ultimately enhancing patient outcomes.

In conclusion, our study demonstrates that the proposed deep CNN-based approach significantly advances the field of ePVS assessment and holds promise for impactful clinical applications. Immediate next steps include multi-center validation and incorporating additional MRI modalities to further enhance the model’s performance and reliability.

## Figures and Tables

**Table 1:** The demographic information of patients with neuropsychiatric examination scores (age, gender, education, MMSE, CDR, and CDRSB).

	AD (N = 8)	MCI (N = 50)	CN (N = 18)	Total (N = 76)
Age	76.2 ± 2	70.3 ± 6.5	67.6 ± 8.9	70.3 ± 7.1
Gender (male/female)	2:6	17:33	6:12	25:51
Education (years)	5.7 ± 3.9	9.8 ± 4.9	12.8 ± 4.8	10.1 ± 5.1
MMSE	22 ± 4.2	26.1 ± 2.7	28.6 ± 1.6	26.3 ± 3.2
CDR	0.6 ± 0.4	0.5 ± 0.1	0.3 ± 0.3	0.4 ± 0.2
CDRSB	3.5 ± 2	1.3 ± 0.9	0.4 ± 0.3	1.3 ± 1.3

**Table 2:** ePVS rating score for a total 456 images (76 subjects × 6 parts) in basal ganglia region.

ePVS Rating (Number of ePVSs)	0 (0)	1 (1–10)	2 (11–20)	3 (21–40)	4 (>40)
Number of images	4	244	138	55	15

**Table 3:** Mean and standard deviation of CNR scores among compared methods and our deep learning-based enhancement method. Scores were measured near the PVS.

	Non-Enhanced	Tophat	Clahe	Laplacian	Deep Learning
CNR	37.8 ± 7.4	42.5 ± 6.0	38.9 ± 7.2	45.9 ± 3.9	113.7 ± 15.1

**Table 4:** Comparison for ePVS quantification results of the non-enhanced, algorithm-based enhanced, and deep learning-based enhanced image.

	MAE	Image-Level Accuracy (%)	Subject-Level Accuracy (%)
Non-enhanced	3.3	74	72
Tophat	3.3	76	72
Clahe	3.1	74	75
Laplacian	3.8	73	63
Deep learning	1.7	88	94

## Data Availability

Data are available from the corresponding author by request.
